# Integrating host transcriptomic signatures for distinguishing autoimmune encephalitis in cerebrospinal fluid by metagenomic sequencing

**DOI:** 10.1186/s13578-023-01047-x

**Published:** 2023-06-19

**Authors:** Siyuan Fan, Xiangyan He, Zhongyi Zhu, Lu Chen, Yijun Zou, Zhonglin Chen, Jialin Yu, Weijun Chen, Hongzhi Guan, Jinmin Ma

**Affiliations:** 1 Peking Union Medical College Hospital, Chinese Academy of Medical Sciences & Peking Union Medical College (CAMS & PUMC), Beijing, 100730 China; 2 Clinical Laboratory of BGI Health, BGI-Shenzhen, Shenzhen, 518083 China; 3 BGI PathoGenesis Pharmaceutical Technology, BGI-Shenzhen, Shenzhen, 518083 China; 4 BGI Infection Pharmaceutical Technology, BGI-Shenzhen, Shenzhen, 518083 China; 5 Beijing Macro & Micro-Test Bio-Tech Co., Ltd., Beijing, 101300 China; 6 College of Life Sciences, University of Chinese Academy of Sciences, Beijing, 100049 China

**Keywords:** Infectious encephalitis, Autoimmune encephalitis, Transcriptomic signatures, Cerebrospinal fluid, Next-generation sequencing (NGS)

## Abstract

**Background:**

The early accurate diagnoses for autoimmune encephalitis (AE) and infectious encephalitis (IE) are essential since the treatments for them are different. This study aims to discover some specific and sensitive biomarkers to distinguish AE from IE at early stage to give specific treatments for good outcomes.

**Results:**

We compared the host gene expression profiles and microbial diversities of cerebrospinal fluid (CSF) from 41 patients with IE and 18 patients with AE through meta-transcriptomic sequencing. Significant differences were found in host gene expression profiles and microbial diversities in CSF between patients with AE and patients with IE. The most significantly upregulated genes in patients with IE were enriched in pathways related with immune response such as neutrophil degranulation, antigen processing and presentation and adaptive immune system. In contrast, those upregulated genes in patients with AE were mainly involved in sensory organ development such as olfactory transduction, as well as synaptic transmission and signaling. Based on the differentially expressed genes, a classifier consisting of 5 host genes showed outstanding performance with an area under the receiver operating characteristic (ROC) curve (AUC) of 0.95.

**Conclusions:**

This study provides a promising classifier and is the first to investigate transcriptomic signatures for differentiating AE from IE by using meta-transcriptomic next-generation sequencing technology.

**Supplementary Information:**

The online version contains supplementary material available at 10.1186/s13578-023-01047-x.

## Background

Encephalitis is the inflammation of the brain parenchyma associated with neurologic dysfunction, which can be life-threatening and contributes to high morbidity worldwide [1]. The symptoms of encephalitis mainly include fever, altered mental status and seizures [2]. The estimated prevalence of encephalitis could reach up to 12.6/100,000 per year (range from 0.07/100,000 to 12.6/100,000) [3]. Encephalitis brought a heavy burden to patients and society, as it was reported that the estimated hospitalization expenses for encephalitis reached $2.0 billion in US in 2010 [4]. Encephalitis can be classified into infectious encephalitis (IE) and autoimmune encephalitis (AE) based on its etiologies. IE was caused by microbial infections including viral, bacterial, fungal and parasitic infections [5]. By contrast, AE was induced by the autoimmunity which was related with the presences of antibodies attacking neuronal intracellular and surface proteins of brain [6]. Previously, most of encephalitis were assumed to result from infections. However, recent studies suggested that the incidence rates of autoimmune encephalitis increased over time and were not significantly different from that of infectious encephalitis [7].

Since the treatments for AE and IE were different, it becomes critical to differentiate AE from IE in the early time to ensure specific treatments. Previous studies indicated that early immunotherapy was important to improve outcomes of patients with AE [8, 9]. The clinical presentations of autoimmune encephalitis kind of overlapped with that of infectious encephalitis, such as fever, seizures and alterations of consciousness [10–13], which increased difficulties in distinguishing them. The traditional diagnosis of autoimmune encephalitis required a variety of tests, such as magnetic resonance imaging (MRI), EEG (electroencephalogram), CSF analysis, neuronal autoantibodies test (NMDAR, LGI1, GAD65, AMPAR, GABAR, and Caspr2 etc.), infection detection in cerebrospinal fluid, examination of inflammatory markers, etc. [14]. However, the combinations of these tests were time-consuming and the sensitivity of diagnosis was low [15]. Therefore, a simpler and highly sensitive approach to distinguish AE from IE is required.

A systematic review of 41 studies on encephalitis showed that more than 50% encephalitis cases were diagnosed with unknown etiology in 26 studies [12]. The etiologies of most of cases with encephalitis were not identified, which suggested the requirements of more advanced diagnostic technologies. In recent years, metagenomic next generation sequencing (mNGS) method has been widely used in the detection of pathogens in clinical samples due to its unbiased, comprehensive, and highly sensitive detection of pathogens [16–19]. mNGS has also been increasingly applied in the diagnosis of suspected infectious encephalitis [20–22], and these clinical studies showed that mNGS could effectively improve the detection of novel, rare or unexpected pathogens, thus enhancing the ability to diagnose infectious encephalitis [23]. Additionally, meta-transcriptomic next generation sequencing (mtNGS), which is a type of RNA-seq, can not only obtain the information of pathogens and microbiome in samples, but also present host gene expression profiles which reveal host responses [24]. Although, there have been many attempts to find markers to distinguish AE from IE from the perspective of clinical characteristics [25–27], the specificity and sensitivity of these markers need further investigation. It has been reported that specific biomarkers can be screened from host and/or pathogen transcription profiles to identify specific pathogen infection [28] or to distinguish different types of pathogen infections [29] or for early diagnosis [30]. So far, there has been no study that takes advantage of transcriptomic profiles by mtNGS to seek biomarkers for distinguishing AE from IE.

In this study, through meta-transcriptomic analysis of CSF, we compared the host responses and microbial diversities between patients with IE and those with AE to explore the differential mechanisms of the occurrence of AE compared to IE and find potential signatures for distinguishing them. At last, we built an efficient classifier, composed with significant DEGs between IE and AE, which has the potential to discriminate the diseases in 2 days, and even in several hours in the future, much faster compared to the traditional approaches that may take several weeks [31].

## Results

### Samples collection and clinical diagnosis

Eighteen patients (male, 61.1%) were definitely diagnosed as AE, according to the approach mentioned in the Materials and Methods section, between 1st June 2019 to 30th July 2019 at Peking Union Medical College Hospital, Chinese Academy of Medical Sciences were recruited. All the patients with AE were CSF anti-NMDAR antibody positive (Cell-based assay). The average age of patients with AE was 22 ± 12.49 years (ranged from 4 to 48 years). Forty-one patients (male, 63.4%) finally were diagnosed as IE at the same hospital and the same period as AE were enrolled in this study, whose average age was 40.39 ± 14.44 (ranged from 12 to 67 years). Cerebrospinal Fluid (CSF) sample was collected from each patient with IE and patient with AE for meta-transcriptomic sequencing (mtNGS).

### RNA-seq quality assessment

The CSF samples were collected from each individual for RNA sequencing. After removing rRNA and low-quality reads, clean reads were produced for samples from patients with AE and patients with IE. Quality control was performed and comparable read depths (4.71 ± 3.34 million reads per sample) were reached for AE and IE samples for the downstream analysis.

### Differential host responses in patients with IE compared to patients with AE

**Fig. 1:**
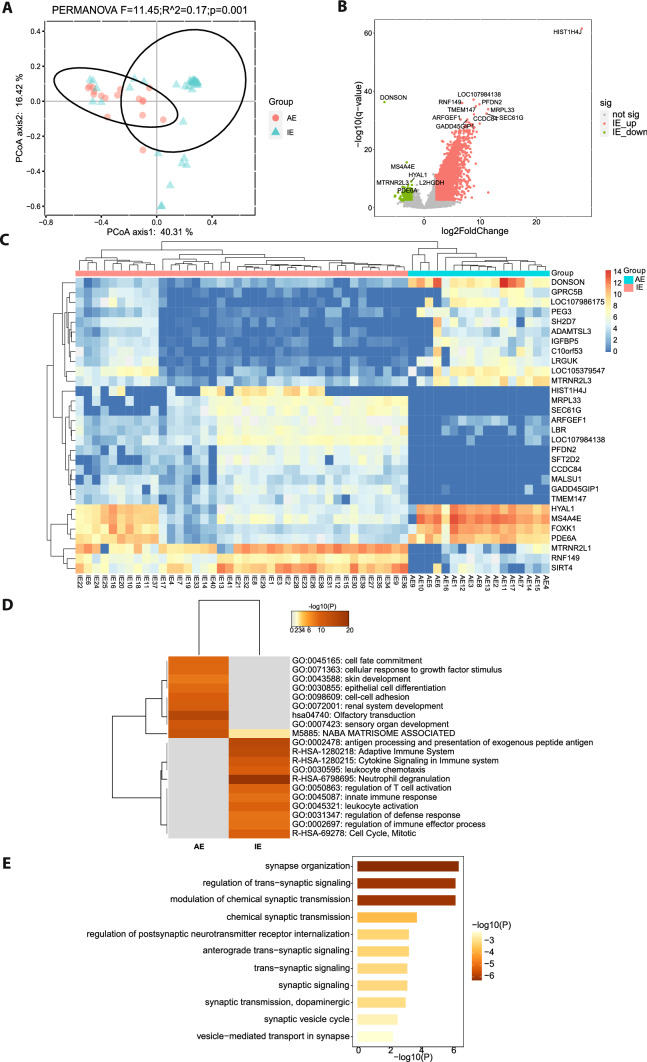
Differential host responses in patients with IE compared to patients with AE. **A** The principal coordinate analysis plot showing the grouping of samples from the two groups based on global gene expression profiles. **B** The volcano plot of DEGs. The significant DEGs are highlighted in red (upregulated genes in IE) or green (down-regulated genes in IE). The unsignificant DEGs are marked in grey. The top 5 distinctly expressed genes are labeled. **C** The heatmap of differential expression of the top 15 significantly upregulated and downregulated genes across all the samples. The genes are ranked by *P* value. The color scale shows different values of log_2_(TPM + 1) which indicates different gene expression levels. The red of the top group bar indicates samples from group IE and the cyan indicates group AE. **D** Gene functional enrichment analysis of DEGs. The heatmap indicates the top 20 representative enriched GO terms. **E** The heatmap showed the enriched GO terms associated with synapse. The color scale indicates the value of − log_10_*P*

To characterize the host responses in patients with different types of encephalitis, the host transcriptomic profiles were compared between patients with AE and patients with IE. A principal coordinate analysis (PCoA) was performed with the host gene expression profiles to examine the clustering of the global transcriptomic profiles of all the samples (Fig. 1A). Two distinct groups were formed with overlaps of few samples between two groups, which suggested the overall obvious difference in transcriptomic profiles between patients with AE and patients with IE. To further analyze the differentially expressed genes (DEGs) which drove the difference, volcano-plot of DEGs was constructed (Fig. 1B). The result showed that 7139 genes (adjusted p < 0.01, |log_2_FoldChange| > 2) were significantly differentially expressed between two groups. The exact details of DEGs were listed in Additional file 1: Table S1. The top 5 significantly up-regulated genes in patients with IE compared to patients with AE, included *HIST1H4J* (Histone H4), *LOC107984138* (Cytochrome P450 2D6-Like), *RNF149* (Ring Finger Protein 149), *PFDN2* (Prefoldin Subunit 2), and *TMEM147* (Transmembrane Protein 147)*,* and the top 5 significantly down-regulated genes including *DONSON* (DNA replication fork stabilization factor), *MS4A4E* (Membrane Spanning 4-Domains A4E)*, HYAL1* (Hyaluronidase 1)*, MTRNR2L3* [MT-RNR2 Like 3 (Pseudogene)], *PDE6A* (Phosphodiesterase 6A). *HIST1H4J* encodes protein Histone H4 which is a core component of histone. DONSON encoding a protein which is a DNA replication fork stabilization factor, plays an important role in maintaining genome stability [32].

To display the differential expression level of the top 30 DEGs across all the samples, a heatmap combined with hierarchical clustering was constructed with the top 15 (ranked by adjusted p value) significant upregulated and downregulated genes of patients with IE versus patients with AE. We found that IE and AE samples were clustered into two groups, indicating that all IE samples had similar expression profiles of the 30 genes which were different from those of AE samples (Fig. 1C). The top 15 upregulated genes in IE were enriched in translation and regulation of cytoskeleton organization (Additional file 2: Fig. S1A). The top 15 upregulated genes in AE may be involved in Meningioma (Additional file 2: Fig. S1B).

Further, functional enrichment analysis of the DEGs (up-regulated genes with log_2_FoldChange > 5, down-regulated genes with log_2_FoldChange < − 2 in group IE versus AE, adjusted P < 0.01, Additional file 1: Table S2) were carried out. The top 20 representative enriched terms were first figured out by Metascape website with the default setting. The upregulated genes in patients with AE versus patients with IE were mainly enriched in 9 terms while these downregulated genes were representatively enriched in 11 terms (Fig. 1D). The upregulated DEGs in patients with AE were mainly associated with olfactory transduction (− log_10_*P* = 15.5), naba matrisome associated (− log_10_*P* = 13.2), sensory organ development (− log_10_*P* = 11.7), renal system development (− log_10_*P* = 10.7), as well as cell differentiation and adhesion. The upregulated DEGs in patients with IE were significantly enriched in pathways such as neutrophil degranulation (− log_10_*P* = 26.87), antigen processing and presentation of exogenous peptide antigen (− log_10_*P* = 14.9) and adaptive immune system (− log_10_*P* = 14.27). Neutrophil degranulation was the most significantly enriched term of these DEGs. These results showed that the genes upregulated in patients with IE versus patients with AE were mainly involved in host immune response, while the genes upregulated in AE versus IE were largely associated with organ development (like olfactory, skin, and renal system) and cell fate (like growth, differentiation, and adhesion).

Since AE was related with the presence of various autoantibodies against proteins localized in or around neuronal synapses [33–36], in addition to the top 20 representative terms described above, we further checked all the functional enrichment terms of these genes to see whether there were some enriched terms associated with synapse. Interestingly, we found that a large number of genes were enriched in terms related with neuronal synapse organization and synaptic signal transmission (Fig. 1E, Additional file 1: Table S3), such as synapse organization (− log_10_*P* = 6.3), modulation of chemical synaptic transmission (− log_10_*P* = 6.1), regulation of trans-synaptic signaling (− log_10_*P* = 6.1), chemical synaptic transmission (− log_10_*P* = 3.2), anterograde trans-synaptic signaling (− log_10_*P* = 3.2), regulation of postsynaptic neurotransmitter receptor internalization (− log_10_*P* = 3.1), synaptic signaling (− log_10_*P* = 3.1), and trans-synaptic signaling (− log_10_*P* = 3). And these terms were all upregulated in patients with AE compared to patients with IE.

### Differential microbial diversity in patients with IE compared to patients with AE

**Fig. 2:**
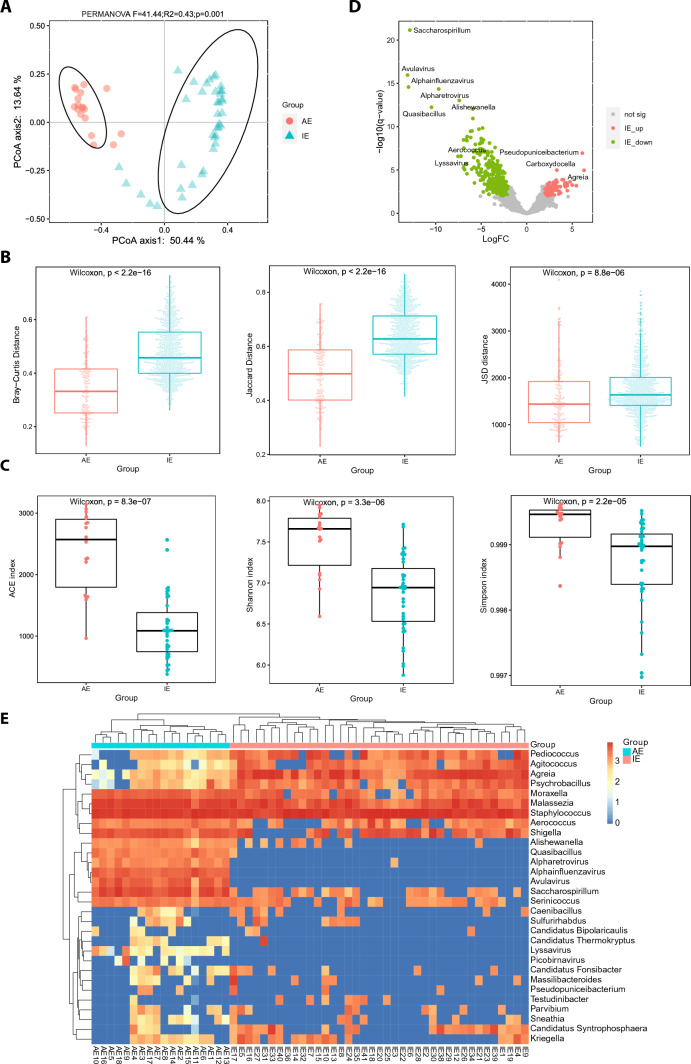
The CSF microbial diversity analysis of patients with IE compared to patients with AE. **A** The principal coordinate analysis plot showing the grouping of samples based on the differences in beta-diversity of CSF microbiome. **B** The beta-diversity analysis of CSF microbiome, including Bray–Curtis distance, jaccard distance and jsd distance. The Wilcoxon rank-sum test was performed to assess the statistical significance. **C** The alpha-diversity analysis of CSF microbiome, including ACE, Shannon and Simpson index. The Wilcoxon rank-sum test was performed to assess the statistical significance. **D** The volcano plot of differentially abundant microbes between the two groups. The significantly differentially abundant microbes are highlighted in red (upregulated microbes in IE) or green (down-regulated microbes in IE). The unsignificant differentially abundant microbes are marked in grey. The top (ranked by adjusted p) differentially abundant microbes are labeled. **E** The heatmap of differential abundances of the top 15 (ranked by log_2_FoldChange, adjusted p < 0.01) significantly upregulated and downregulated genera across all the samples

To examine the diversity of microbes detected in CSF samples of patients with IE and patients with AE, a PCoA plot was generated based on the differences in beta-diversity of CSF microbiome among all the samples. The figure (Fig. 2A) shows that the samples of patients with AE were distinctly separated from those of patients with IE and all samples were clustered into two isolated groups. This indicated that there was significant difference in microbial diversity between patients with IE and patients with AE. To compare the dissimilarity of composition of microbes in CSF among samples from patients with IE and among samples from patients with AE, beta-diversity indices including Bray–Curtis distance, Jaccard distance and Jensen-Shannon divergence (JSD) were analyzed in the two groups (Fig. 2B). Larger values of Bray–Curtis distance (p < 2.2e−16), jaccard distance (p < 2.2e−16) and JSD (p = 8.8e−06) in patients with IE, suggested that there was a greater difference in CSF microbial diversity among patients with IE than that among patients with AE. This may be attributed to that the dominant infectious pathogens, such as virus, bacteria and fungus, varied among different CSF samples from patients with IE, and these distinct pathogens may have different impacts on CSF microbial composition. To further determine diversity of microbes within each CSF sample from these two groups, alpha-diversity indices including ACE, Shannon and Simpson were analyzed (Fig. 2C). ACE index indicates richness while Shannon or Simpson index represents diversity of microbes in samples. The overall diversity of microbes decreased in patients with IE compared to patients with AE, since the values of ACE (*P* = 8.3e−07), Shannon (*P* = 3.3e−06) and Simpson (*P* = 2.2e−05) indices in patients with IE were significantly less than that in patients with AE. A lower alpha-diversity in CSF samples of patients with IE can be due to that the infectious pathogens were dominated in the microbiomes of CSF from patients with IE, which decreased the relative abundances of other microbes.

We then investigated the differences in abundances of microbes in CSF at genus level between IE group and AE group. Total 3620 microbial taxa (at genus level) were detected in these two groups. The volcano-plot (Fig. 2D) showed the differentially abundant microbes (at genus level) in CSF between the two groups. There were 416 genera (adjusted p < 0.01, |log_2_FoldChange| > 2) which had significantly different abundances in patients with IE versus patients with AE, and among them, 92 and 324 genera showed upregulated and downregulated abundances in group AE compared to group IE, respectively (Additional file 1: Table S4). *Pseudopuniceibacterium* (*P* = 1.48e−09), *Carboxydocella* (*P* = 3.19e−07), and *Agreia* (*P* = 3.37e−07) were the top three significantly upregulated genera in group IE. *Saccharospirillum* (*P* = 2.23e−25), *Avulavirus* (*P* = 6.84e−20), *Alphainfluenzavirus* (*P* = 2.49e−18), *Alpharetrovirus* (*P* = 5.61e−18) and *Alishewanella* (*P* = 1.47e−16) were the top five upregulated genera in patients with AE.

To further examine the abundances of microbes at genus level across all the samples, a hierarchical clustered heatmap was constructed according to the abundances of the top 15 (ranked by log_2_FoldChange, adjusted *P* < 0.01) significantly up-regulated and down-regulated microbial genera (Fig. 2E). All AE samples were clustered together into one group, as the same as all IE samples. According to the abundance levels of microbials, the 30 microbial genera were clustered into 3 distinct groups. The abundance levels of microbials clustered into the group 2 showed significant upregulation in AE group versus IE group. The microbial taxa of group 2 included *Alishewanella*, *Quasibacillus*, *Alpharetrovirus*, *Alphainfluenzavirus*, *Avulavirus*, *Saccharospirillum* and *Serinicoccus* genera, which interestingly comprised three viral genera that showed lower abundances in almost 100% patients with IE compared to each of patients with AE. This means that *Alpharetrovirus*, *Alphainfluenzavirus*, and *Avulavirus* genera might have great potentials to act as biomarkers to differentiate patients with IE from patients with AE.

### Interactions between host genes involved in neutrophil degranulation as well as genes associated with synaptic transmission and signaling and CSF microbes

**Fig. 3:**
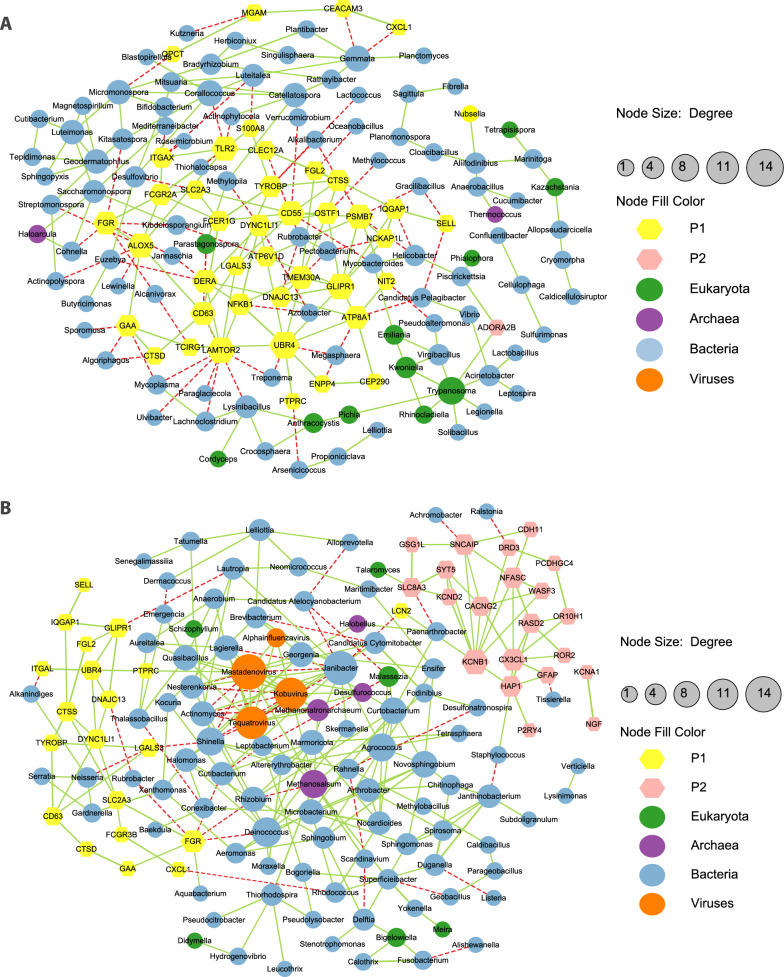
Network analysis of the correlations of DEGs involved in neutrophil degranulation with CSF microbes and microbe-microbe interactions. **A** The interactions detected in patients with IE. **B** The interactions detected in AE cases. Each node represents one human gene or one microbial genus and the corresponding gene or microbe names are labeled. The yellow (P1) and magenta (P2) nodes indicate human genes involved in neutrophil degranulation and synaptic transmission and signaling respectively. The green, purple, blue and orange nodes indicate microbial genera belong to eukaryota, archaea, bacteria and viruses respectively. Red edges indicate negative correlations and green edges indicate positive correlations. The width of edge indicates interaction weight, and increases with the rise of weight. The size of node indicates the number of connections with other nodes, namely, the degree

As described above, functional enrichment analysis of DEGs showed that neutrophil degranulation pathway was the top significantly enriched pathway and 60 genes involved in this term were upregulated in patients with IE. In contrast, 49 genes associated with synaptic transmission and signaling were upregulated in patients with AE. To investigate the interactions between genes enriched in these two pathways and the microbes, the correlations between the 109 genes and 3620 detected microbial taxa (at genus level) were analyzed in patients with IE and patients with AE respectively by spearman's rank correlation coefficient. The genes and microbes (microbial taxa at genus level) with an correlation coefficient |rho| ≥ 0.6 were selected and used for the correlation-based network analysis. The correlation networks from group IE and AE showed significantly different structures (Fig. 3A, B). The network from group IE consisted of 42 host genes including 41 genes involved in neutrophil degranulation and one gene related to synaptic transmission and signaling, and 100 microbial genera (including 87 bacterial, 2 archaea and 11 eukaryotic genera), while that from group AE were comprised of 20 genes associated with neutrophil degranulation and 21 genes involved in synaptic transmission and signaling, and 101 microbial genera (including 87 bacterial, 4 archaea, 6 eukaryotic and 4 viral genera). In addition, there was a total of 63 direct correlations between 36 host genes and 46 microbial genera in group IE (Fig. 3A, Additional file 1: Table S5), while only a total of 19 direct interactions between 13 genes and 18 microbes were found in group AE (Fig. 3B, Additional file 1: Table S6). Specifically, in patients with IE, 35 genes from neutrophil degranulation pathway (such as *ALOX5*, *FGR*, *IQGAP1*) had direct and negative correlations with 42 microbial genera including 41 bacteria (such as *Lewinella and Cohnella*) and one fungus (*Parastagonospora*), and one gene (*ADORA2B*) from synaptic transmission and signaling were observed to be positively correlated with one microbial genera *Trypanosoma*. In comparison to patients with IE, 8 genes (such as *CXCL1*, *LCN2*, *FGR*) from neutrophil degranulation pathway were directly associated with 12 microbial genera (including 11 negative and 2 positive correlations) and 5 genes from synaptic transmission and signaling (*DRD3*, *SNCAIP*, *GFAP*, *HAP1*, *SLC8A3*) were observed to be correlated with 6 microbes (including 4 negative and 2 positive associations) in patients with AE. These indicated that a smaller number of host genes directly interacted with microbes in patients with AE compared to patients with IE. In addition, interestingly, the microbial genera associated with genes in neutrophil degranulation pathway in patients with IE were remarkably different from that found in patients with AE except for one shared genus which is *Rubrobacter*. The interactions between genes in synaptic transmission and signaling and microbes were distinct in patients with IE and patients with AE, since no shared genes and microbes were found between them. Only one gene involved in synapse organization and signaling were presented in the correlation network in IE group while 21 genes related with that showed 36 positive inner associations in AE group, indicating that these genes were specifically co-expressed in AE groups. These suggested that the host gene expression patterns and host-microbe interactions were different between the two groups, which supports the above results that patients with IE and patients with AE had distinct host responses. And noteworthy, all the microbes showed negative correlations with genes related to neutrophil degranulation. These suggested that neutrophil degranulation induced by pathogen infections in patients with IE may decrease the abundance of microbials such as *Lewinella*, *Cohnella*, *Helicobacter*, *Pectobacterium*, *Euzebya*, and *Oceanobacillus*, or that these microbials may have a negative impact in neutrophil degranulation.

### Classifiers to distinguish AE from IE

**Fig. 4:**
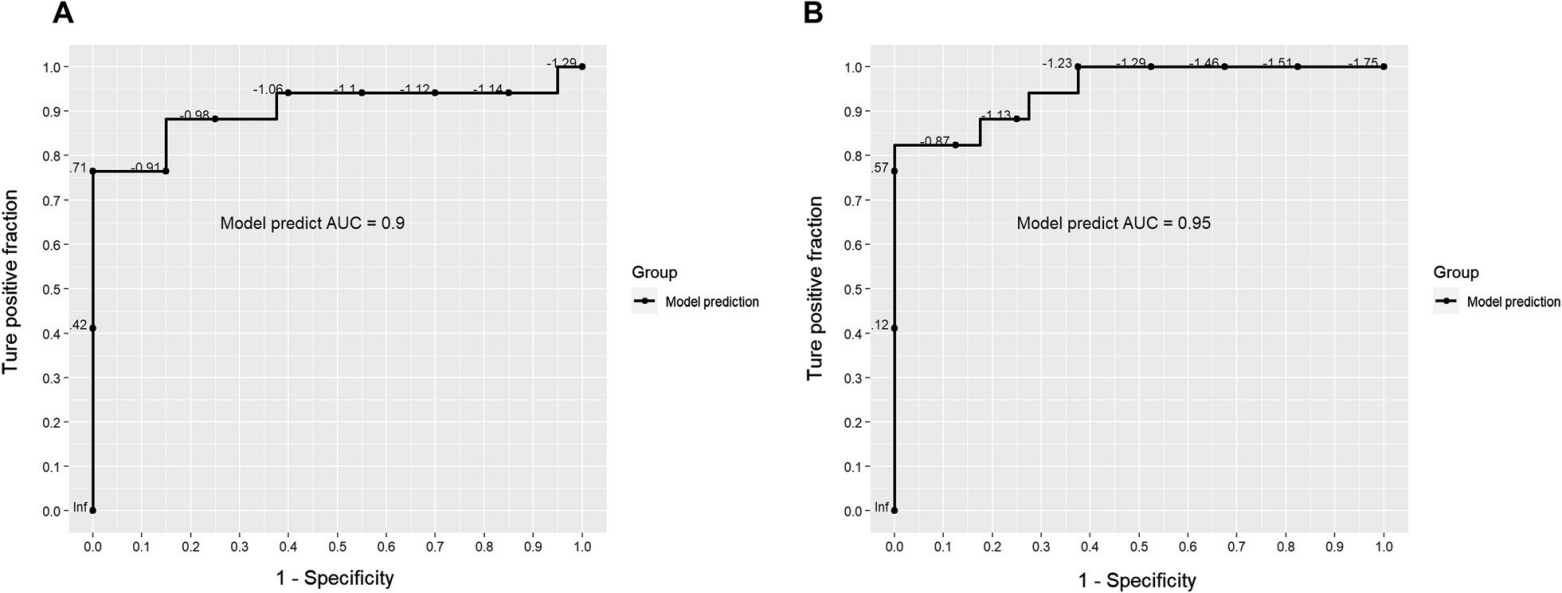
The receiver operating characteristic curve showing the performance of host transcriptomic signatures to distinguish AE from IE. **A** The ROC curve indicates the performance of 1-gene classifier. **B** The ROC curve indicates the performance of 5-gene classifier

As mentioned above, many genes were significantly differentially expressed between patients with IE and patients with AE. To discriminate AE from IE, the top 1000 significant DEGs were selected and applied for the screen of markers. The classifiers were developed by Lasso regression, and ten-fold cross validation were used to produce AUC (area under the ROC curve) value of each classifier. The classifier with one host gene signature *MS4A4E* (Membrane Spanning 4-Domains A4E) had an AUC of 0.9 (Fig. 4A). With the increase in the number of gene signatures, the performance of classifier improved. The classifier with five host gene signatures including *MS4A4E*, *TGS1* (Trimethylguanosine Synthase 1), *POLE3* (DNA polymerase epsilon 3), *EAPP* (E2F Associated Phosphoprotein), and *RASAL3* (RAS Protein Activator Like 3) had an AUC of 0.95 (Fig. 4B). The best host gene signature *MS4A4E* that encodes protein Membrane Spanning 4-Domains A4E, which may be related with Alzheimer’s disease [37]. *MS4A4E* was significantly (*P* adjust = 2.56E−16) upregulated in patients with AE compared to patients with IE, which may also potentially play a role in the occurrence of AE. Interestingly, except *MS4A4E* gene, the other four genes (*TGS1, POLE3, EAPP, RASAL3*) were all upregulated in patients with IE. TGS1 is also known as PRIP-interacting protein with methyltransferase domain (PIMT) which interacts with coactivator peroxisome proliferator-activated receptor-interacting protein (PRIP), and may be involved in gene transcription regulation [38, 39]. POLE3 is involved in DNA replication and lymphocyte (T and B) development in mice [40]. RASAL3 plays an important role in survival of peripheral naive T cells in mice [41].

## Discussion

As the treatments for AE and IE are different, distinguishing AE from IE in the early time is essential for specific treatment. So far, it is still a big challenge for doctors to differentiate AE from IE due to the lack of effective and comprehensive signatures. The methods used to discriminate them yet require a variety of tests, with low sensitivity. Meta-transcriptomic sequencing can provide information on host response and microbial composition in the sample at the same time. In this study, we identified significant differences in host response and microbial composition of CSF between patients with IE and patients with AE, as well as the interactions between neutrophil degranulation-related genes or synaptic transmission and signaling-associated genes and microbes of CSF. On the base of these differences, we further developed a classifier composed of 5 host genes to distinguish AE from IE, which has an outstanding performance with an AUC of 0.95. To the authors’ knowledge, this is the first study to investigate biomarkers for differentiating AE from IE from the perspective of host transcriptomic profiles by using mtNGS technology.

According to the functional enrichment analysis of DEGs, the most significantly enriched term was neutrophil degranulation, which was upregulated in IE cases versus AE cases. The neutrophils, also termed polymorphonuclear leukocytes (PMNs), accounting for about 70% of all leukocytes essential for the innate immunity, form the first line of defense against pathogen infections especially for bacteria and fungi [42]. One mechanism of neutrophils killing microbials is degranulation by releasing various microbicidal components such as elastase, myeloperoxidase (MPO) and defensins [43]. Since IE is caused by pathogen infection which would induce neutrophils infiltration and activate neutrophils, it is rational to see upregulations of genes involved in neutrophil degranulation. Similar phenomenon was observed by Michael et al. [27] who found that MPO level in CSF was higher in patients with IE than that in patients with AE, and neutrophils were more frequently detected in patients with IE versus patients with AE. It was also reported that the infection of Japanese encephalitis virus (JEV) which causes human encephalitis, activated neutrophil degranulation in mice [44]. In addition, SARS-Cov2 infection significantly increased the expression of genes involved in neutrophil degranulation in the lungs of rhesus macaques [45]. These suggest that neutrophil degranulation is common in response to pathogen infections. The neutrophil degranulation pathway was the top significantly enriched pathway and upregulated in patients with IE, which suggests that neutrophil degranulation also played an essential role in defensing pathogen infections in brain parenchyma.

The significantly downregulated genes in IE compared to AE were enriched in olfactory transduction. It was reported that the increased expression of cathepsins (cathepsins A, D, and S) in olfactory bulbs of mice with induced autoimmune encephalomyelitis may contribute to olfactory dysfunction in these mice [46]. The Cathepsin S may inhibit the binding of the olfactory transcription factor (Olf-1) to the target genes (including olfactory receptors), and its overexpression significantly downregulated the transcription of olfactory receptor such as *OR10AD1*, *OR10H3* and *OR56A4* [47]. Olfactory receptor family was critical for odor perception [48]. We found that cathepsins D and S were significantly transcribed in CSF of patients with IE versus patients with AE (Additional file 1: Table S1), and a lot of olfactory receptors were significantly downregulated in patients with IE versus patients with AE, such as *OR10AD1*, *OR10H3, OR3A3*, *OR2F1*, *OR10G2*, *OR10H3*, *OR7E24*, *OR1J2*, *OR7D4*, *OR1N1*, *OR52I2*, *OR5D14*, *OR5L1*, *OR5M8*, *OR5AR1*, *OR4D5*, *OR6Q1*, *OR52B2*, *OR4C3*, *OR51V1*, *OR52L1*, *OR2AG2*, and *OR1B1*. Landis et al. [49] showed that functional anosmia happened to three patients with herpetic meningoencephalitis and the dysfunctions were not recovered significantly even after 3 years. Consistently, two patients with SARS-CoV-2-associated encephalitis/meningitis were reported with loss of smell [50, 51]. Besides, about 47.85% COVID‐19 patients showed olfactory dysfunction, which was concluded from 83 studies [52]. These results may suggest that IE may have a greater impact on olfactory function than AE. Or olfactory pathway may play a role in the entry or spread of pathogens into brain, as this was also questioned by Landis et al. [49].

The genes related with synapse organization and signaling were upregulated in patients with AE compared to patients with IE. This may be due to that the autoantibodies produced in patients with AE target proteins localized in neuronal synapse (synaptic membrane or space), such as NMDAR, LGI, AMPAR, GABA receptors, mGluR5, D2R, and Neurexin-3α, causing these proteins crosslinking and internalization and affecting synaptic signal transmission [34], which may induce the host response to repair the synapse organization and recover the synaptic signaling. Besides, the presence of Anti-NMDA receptor antibodies is a common cause of AE [12, 53]. It was reported that these autoantibodies may lead cross-linking and internalization of NMDA receptors (NMDAR) resulting in impaired NMDAR-mediated functions such as the decrease in synaptic NMDAR-mediated currents [54]. In this cohort of patients with AE, the CSF samples from patients with AE were tested anti-NMDAR antibodies positive, which may explain why the genes involved in the positive regulation of NMDA glutamate receptor activity were downregulated in patients with AE.

The microbial diversity of CSF was compared between patients with IE and patients with AE, which may be astonishing since the CSF of healthy person has been thought to be sterile for a long time. However, in recent years, taking advantage of unbiased next generation sequencing technology, a study showed that there was a community of DNA viruses which mainly comprised bacteriophages in CSF of healthy people [55]. Since CSF microbiome profiles in healthy individuals were not determined yet, therefore we set strict controls to ensure the same treatment for AE and IE samples to eliminate the possible impact of other unrelated factors. Besides, RNA sequencing was applied in this study to analyze microbial composition, which also enhanced the reliability of the results by presenting more active microbes compared to DNA sequencing. Hence, the differentially abundant microbes discovered in this study should be meaningful. The significant difference in microbial diversity between group IE and AE, suggested that microbial composition or at least the microbial RNA levels in CSF of encephalitis patients were associated with the types of encephalitis.

As presented above, threefold microbial genera in patients with IE than patients with AE were found to be negatively correlated with genes in neutrophil degranulation. This can be explained by that pathogen infection recruit neutrophils and induce neutrophil degranulation, which may reduce the abundances of some microbes. Besides, it’s interesting that the associated microbes with genes involved in neutrophil degranulation in IE were completely different from that found in AE, which may be due to the distinct divergence of microbial composition between patients with IE and patients with AE, as presented by PCoA plot (Fig. 2A).

Massive efforts have been carried out to find features and predictors or markers to distinguish IE from AE in clinic [7, 10, 15, 27, 56, 57], but most of the attempts were from the perspective of clinical characteristics which require a variety of tests such as microbial culture, blood test (cell count, serum test), various imaging tests (MRI), autoantibody detection and etc. Huang et al. [56] showed that patients with AE tended to be more related with symptoms like memory deficits, involuntary movement as well as seizures and were more often found with hippocampus lesions, while patients with IE presented higher positive rates in Pandy tests and had lower counts of serum erythrocyte and platelet. They suggested that the combination of these features may provide a clue for differentiating AE from IE. However, these tests were time-consuming and the accuracy required further determination. Graus et al. [8] developed a clinical algorithm on the base of clinical features, MRI, CSF analysis, and EEG to identify AE. Then Wagner et al. [15] applied this algorithm to differentiate their cohort of encephalitis patients, and found that the sensitivity and specificity was very low for the diagnosis of AE and the accuracy was not only time-dependent but also relied on the microbiological tests such as PCR for the detection of various virus and cultures for bacteria. It was also reported that there was significant difference in CD4:CD8 cells ratio in CSF between anti-*N*-methyl-d-aspartate receptor AE and herpes simplex virus encephalitis [58], while this marker need verified widely in more other types of AE and IE. As mentioned above, mNGS has been increasingly used in the diagnosis of suspected infectious encephalitis to find the exact pathogens to ensure specific treatments [20–22]. In addition to pathogen identification, RNA-based mtNGS can provide information on host response and microbial diversity. In this study, we also tried to take advantage of RNA-based mtNGS to discover signatures from the view of differences in host gene expressions to enhance the discriminability. Markedly different markers were found compared to previous studies. And interestingly, the best host gene signature *MS4A4E* that encodes protein Membrane Spanning 4-Domains A4E, which may be related with Alzheimer’s disease [37]. *MS4A4E* was significantly (p adjust = 2.56e−16) upregulated in patients with AE compared to patients with IE, which may also potentially play a role in the occurrence of AE. Besides, the marker *RASAL3,* which was significantly upregulated (62-fold change, adjusted p = 4.6e−25) in patients with IE versus patients with AE, played an important role in modulating the number and function of natural killer T (NKT) cells in mouse liver [59]. It was reported that NKT cells contributed to the defense of host against bacterial and fungal infections [60, 61], which may explain why RASAL3 was more expressed in patients with IE. In summary, this study indicates that the use of mtNGS will facilitate the differentiation of AE from IE. So far, many hospitals in China have established their own local next-generation sequencing platforms which were applied for pathogen detection by mNGS. Hence, it will be easy to transfer the signatures found in this study to clinical application and improve the diagnostic capability. Besides, five host gene signatures showed excellent performances, suggesting that RT-qPCR or serum test based on the five host gene signatures may be applied first in clinical practice and may achieve accurate diagnosis in 2 to 3 h for the differentiation of IE from AE.

It was reported that lactate concentration was a good predictor for discriminating bacterial encephalitis from other types of encephalitis, and increased number of mononuclear cells to some extent can predict viral infections [57]. Due to the limited number of CSF samples from patients with IE, it is not rational to separate them into different groups according to different infectious pathogens like bacteria, virus, fungus and parasite in this study. Further work can be done by comparing differences in host response and microbial diversity of CSF between different types of infectious encephalitis, to find signatures to distinguish viral encephalitis from bacterial or fungal or parasitic encephalitis, or even differentiate them at the genus or species level.

## Conclusion

There were significant differences in host response and microbial diversity in CSF between patients with AE and IE unraveled by meta-transcriptomic sequencing. Some genes upregulated in IE patients were mainly related with neutrophil degranulation, antigen processing and presentation of exogenous peptide antigen and adaptive immune system, while those upregulated in AE patients were largely involved in sensory organ development such as olfactory transduction, as well as synaptic transmission and signaling. Comparison of transcriptomic profiles between patients with AE and those with IE by meta-transcriptomic sequencing could be an efficient way to find specific and effective biomarkers for distinguish AE from IE. A classifier developed with 5 host genes showed outstanding performance for distinguishing AE from IE, with an AUC of 0.95.

## Materials and methods

### Study design and ethics

In order to compare differences in host response and microbiome diversity in brain parenchyma between patients with autoimmune encephalitis (AE) and infectious encephalitis (IE) to find biomarkers for differentiate AE from IE, patients confirmed with AE and IE were recruited. Patients who presented with symptoms of abnormal (psychiatric) behavior, cognitive dysfunction, decreased level of consciousness, or seizures, showed pleocytosis, and were tested positive for cerebrospinal fluid (CSF) anti-NMDAR antibody (determined by a cell-based assay), as well as met the diagnostic criteria for anti-NMDAR encephalitis proposed by Graus et al. [8], were included in the autoimmune encephalitis (AE) group. To rule out viral encephalitis, PCR testing for herpes simplex virus types 1 and 2 in cerebrospinal fluid or metagenomic next-generation sequencing (mNGS) in CSF were performed. Furthermore, patients with herpes simplex virus-induced anti-NMDAR encephalitis and those who cannot be definitely diagnosed as AE were excluded from the study. The patients diagnosed with AE at Peking Union Medical College Hospital, between 1 June and 30 July 2019 were all recruited. Meanwhile, the patients that had symptoms (such as decreased level or loss of consciousness, dizziness, cognitive dysfunction, seizures, headache and fever) and clinical features of infection, in combination with the detection of encephalitis-associated pathogens in CSF, and met the criteria suggested by the International Encephalitis Consortium [1], were diagnosed as IE and were enrolled in this study. Besides, the patients with IE simultaneously tested positive for common neuronal autoantibodies (NMDAR) and those who cannot be definitely diagnosed as IE were excluded.

This study was approved by the institutional review board (JS-2182) at Peking Union Medical College Hospital, Chinese Academy of Medical Sciences.

### Sample collection and meta-transcriptomic sequencing

Cerebrospinal Fluid (CSF) sample (1.5 to 3 mL) was collected from each AE and IE patients. RNA of these samples was extracted respectively using QIAamp Viral RNA Kits (Qiagen). rRNA was removed with KAPA RiboErase Kit (HMR) (Roche) before the synthesis of cDNA and the construction of DNA libraries. The DNA libraries of CSF samples from AE and patients with IE were constructed and then sequenced by MGISEQ-2000 platform (BGI) or Illumina NextSeq 500 platform (Illumina). Two platforms were used to extend applicability of results produced in this study.

### Sequencing data processing

The raw sequencing data were first filtered by fastp (version 0.20.0) [62] and the quality control was assessed by FastQC (version 0.11.9) [63]. Then the ribosome RNA data were removed by SortMeRNA (version 4.3.3) [64] by six silva rRNA database (silva-bac-16s-id90, silva-bac-23s-id98, silva-euk-18s-id95, silva-euk-28s-id98, silva-arc-16s-id95 and silva-arc-23s-id98). Some residual ribosome RNA data were further completely removed manually. The obtained non-rRNA sequencing data were then mapped to human hg19 reference, and RSEM [65] (version: 1.2.22; parameter: --forward-prob 0.5 --paired-end -p 8, bowtie2 version 2-2.2.5; parameter: bowtie2 -q --sensitive --dpad 0 --gbar 99999999 --mp 1,1 --np 1 --score-min L, 0, -0.1 -I 1 -X 1000 --no-mixed --no-discordant -p 8 -k 200) was used to obtain the raw abundance counts of each gene and also TPM values of each sample. Besides, non-human sequences derived from HISAT2 (version 2.1.0) [66] were used for microbiome analysis. Kraken2 (version 2.0.7-beta) [67] with in-house built Refseq database (Refseq release 200, May 4, 2020) was used to produce the microbial abundance matrix. And then, Bracken [68] was applied to adjust kraken2 results to a more precise and qualified values at genus level.

### Differential gene expression analysis and gene functional enrichment analysis

The differentially expressed genes (DEGs) of the host were obtained by R package DESeq2 [69]. The gene which had less than 10 total mapping reads from all samples or was detected in less than 5 samples was filtered out. The fitType of Deseq was set to “local” and FDR was calculated to adjust p-value. For the obtained differentially expressed gene list, the standard with FDR < 0.01 and log_2_FoldChange > 2 or log_2_FoldChange < − 2 was used to filter genes up-regulated and down-regulated in encephalitis samples. A heatmap combined with hierarchical clustering was constructed with the top 15 (ranked by adjusted p value) significant upregulated and downregulated genes by package Pheatmap (version 1.0.12) [70] in R. The DEGs (FDR < 0.01, log_2_FoldChange > 5 or log_2_FoldChange < − 2) were then supplied to Metascape [71] to do pathway & process enrichment analysis with default parameters. Since 6515 upregulated and only 624 downregulated genes were found, a higher threshold (log2FoldChange > 5) for upregulated genes was used to do functional analysis, which was to minimize the possible false positive effect on DEGs analysis by DESeq model due to a higher number of IE patients compared to AE patients, and also to focus on the most significantly differentially expressed genes.

### Microbiome analysis

Due to the natural sparsity of the microbial abundance matrix, the differential abundance analysis of microbial taxa (at genus level) was performed by R package MetagenomeSeq [72]. This package utilizes normalization to deal with biases in annotations and zero-flatted Gaussian distribution to deal with the impact of sequencing depth. On this basis, the differences of microbial abundances were identified using linear models. The microbial genus which had counts in less than 5 samples was removed. We used wrenchNorm functions to differentiate phenotype from cumNorm. A volcano plot was used to present the differentially abundant genera. The genera with − log_10_(q-value) > 2 and Log_2_FoldChange > 2 were marked as up-regulated taxa in patients with IE, and the genera with − log_10_(q-value) > 2 and Log_2_FoldChange < − 2 was marked as down-regulated taxa in patients with IE. For the analysis of microbial diversity, we used R package Fossil [73] and VEGAN [74] to analyze α-diversity, and R package Philentropy [75] to analyze β-diversity. PCoA (principal co-ordinates analysis) was conducted by R package *vegan* [74]. The heatmap combined with hierarchical clustering was developed with the top 15 (ranked by log_2_FoldChange) significantly upregulated and downregulated microbes (at genus level) by R package Pheatmap [70] in R.

### Host-microbe correlation network construction

Spearman correlation analysis was conducted to preliminarily figure out the correlations of host genes and microbes. The interactions (including gene-microbe, gene–gene, and microbe-microbe) with correlation coefficient *r*_s_ > 0.6 or r < − 0.6 were selected out, and then these associated genes and microbes were used for the construction of SPIEC-EASI network using r-packet SpiecEasi [76]. For the network construction, we set the parameters as method = 'MB', lambda.min.ratio = 1e−2, nlambda = 20, pulsa.params = list, and Rep. num = 50. R package igraph [77] was used to extract network nodes and edges, and package Intergraph [78] and ggnetwork [79] were used to annotate and show the interaction relationship. To better show the interactions, the output data including correlated genes, microbes, interaction relationship and weight, were imported into cytoscape [80] to generate the final correlation networks.

### The development of classifier

Since the dimensionality of features of variables was huge, therefore L1 regularized Lasso regression was used to achieve feature shrinkage. Specifically, we used the R-package *GLMNET* [81] to fit the generalized linear model to conduct feature screening and modeling for host genes. The model parameter family was set to “binomial”, since the dependent variables were binary variables [autoimmune encephalitis (AE) or not]. The lambda value was set to 50, and ten times cross validation were conducted to produce AUC values of each model according to the test set. The model with the highest AUC and the minimum number of genes was chosen as the best model, and the genes were regarded as potential markers to differentiate IE from AE. After determining the model, the coefficient of variables and optimal variables of the model were obtained.

### Statistical analysis

Comparison of differences in the global transcriptional profiles and microbial diversity between AE and IE in PcoA plot were assessed by Permutational multivariate analysis of variance (PERMANOVA). The differences of gene expressions between group IE and AE were examined by R package DESeq2. The significance of differences in abundances of microbial taxa (at genus level) between the two groups was valued by R package MetagenomeSeq. The comparisons of microbial diversity between groups were assessed by Wilcoxon rank sum test. The correlations of gene-microbe, gene–gene, and microbe-microbe were tested by Spearman correlation analysis. The performances of models were assessed by AUC values.

## Supplementary Information

**Additional file 1: Table S1.** Differentially expressed host genes in IE versus AE. **Table S2.** Differentially expressed genes for functional enrichment analysis. **Table S3.** Functional enrichment analysis of DEGs and the enriched terms. **Table S4.** Differentially abundant microbes (at genus level) in IE versus AE. **Table S5.** Correlations of differentially expressed genes involved in neutrophil degranulation or synaptic transmission and signaling with CSF microbes in IE patients. **Table S6.** Correlations of differentially expressed genes involved in neutrophil degranulation or synaptic transmission and signaling with CSF microbes in AE patients.

**Additional file 2: Figure S1.** The functional enrichment analysis of DEGs. (A) The functional enrichment of the top 15 upregulated genes in IE compared to AE. (B) The top 15 upregulated genes in AE compared to IE may be associated with Meningioma.

## Data Availability

All data are available in the main text or the Additional files. All non-human sequencing data generated in this study are available in the CNGB Nucleotide Sequence Archive (CNSA: https://db.cngb.org/cnsa; accession number CNP0003472).

## Abbreviations

IE: Infectious encephalitis
AE: Autoimmune encephalitis
CSF: Cerebrospinal fluid
NGS: Next-generation sequencing
AUC: Area under the ROC curve
DEG: Differentially expressed gene
PCoA: Principal coordinate analysis
mtNGS: Meta-transcriptomic next-generation sequencing
