# Supplementary material for: Integrating host transcriptomic signatures for distinguishing autoimmune encephalitis in cerebrospinal fluid by metagenomic sequencing
Source: Cell Biosci. 2023 Jun 19;13:111. doi: 10.1186/s13578-023-01047-x (PMC10278324; doi:10.1186/s13578-023-01047-x)
Supplement: Supplementary file 2 — Additional file 2: Figure S1. The functional enrichment analysis of DEGs. (A) The functional enrichment of the top 15 upregulated genes in IE compared to AE. (B) The top 15 upregulated genes in AE compared to IE may be associated with Meningioma. [file 13578_2023_1047_MOESM2_ESM.docx]

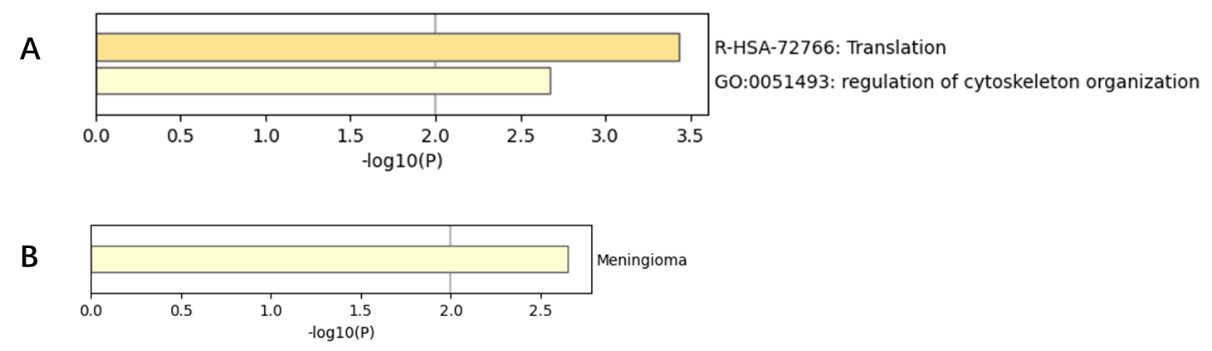


**Figure S1**. **The functional enrichment analysis of DEGs.** (A) The functional enrichment of the top 15 upregulated genes in IE compared to AE. (B) The top 15 upregulated genes in AE compared to IE may be associated with Meningioma.
